# CRISPR-based gene replacement reveals evolutionarily conserved axon guidance functions of *Drosophila* Robo3 and *Tribolium* Robo2/3

**DOI:** 10.1186/s13227-017-0073-y

**Published:** 2017-06-01

**Authors:** Timothy A. Evans

**Affiliations:** Department of Biological Sciences, University of Arkansas, Fayetteville, AR 72701 USA

**Keywords:** Axon guidance, Longitudinal pathways, *Drosophila*, *Tribolium*, Slit, Roundabout, CRISPR

## Abstract

**Background:**

Axon guidance receptors of the Roundabout (Robo) family regulate a number of axon guidance outcomes in bilaterian animals in addition to their canonical role in Slit-dependent midline repulsion. In the fruit fly *Drosophila melanogaster*, three Robo paralogs (Robo1, Robo2, and Robo3) each have specialized roles in regulating midline crossing and the formation of longitudinal axon pathways in the embryonic ventral nerve cord. The number of *robo* genes differs in other insects, and it is unknown whether the roles and/or signaling mechanisms of *Drosophila* Robos are shared in other insect species. To directly compare the axon guidance activities of Robo receptors in *Drosophila* and the flour beetle *Tribolium castaneum,* I have used a CRISPR/Cas9-based approach to replace *Drosophila robo3* with *Tribolium robo2/3*.

**Results:**

I show that when expressed from the *robo3* locus in *Drosophila* embryos, *Tribolium* Robo2/3 (TcRobo2/3) protein is properly translated and localized to axons, where it reproduces the normal expression pattern of *Drosophila* Robo3. In embryos expressing *TcRobo2/3* in place of *robo3*, two distinct subsets of longitudinal axons are guided properly to their normal positions in the intermediate neuropile, indicating that TcRobo2/3 can promote Robo3-dependent axon guidance decisions in developing *Drosophila* neurons.

**Conclusions:**

These observations suggest that the mechanism by which *Drosophila* Robo3 promotes longitudinal pathway formation is evolutionarily conserved in *Tribolium*, where it is performed by TcRobo2/3. The CRISPR/Cas9-based gene replacement approach described here can be applied to comparative evolutionary developmental studies of other *Drosophila* genes and their orthologs in other species.

## Background

### Three-dimensional architecture of the insect ventral nerve cord

During development of the nervous system, neural circuits form as axons and dendrites follow directional cues which guide them to specific regions of the central nervous system (CNS), where local contact-dependent cues promote the formation of synaptic connections. In the insect ventral nerve cord, a stereotypical three-dimensional arrangement of axons and dendrites forms within each segmentally repeated neuromere [1, 2]. In the fruit fly *Drosophila melanogaster*, where the underlying genetics has been best studied, the medial–lateral and dorsal–ventral position of dendrites, axon terminals, and longitudinal axon pathways within each neuromere is specified by a combination of extracellular guidance cues including Slit and semaphorin ligands, and their neuronal receptors of the Roundabout (Robo) and Plexin families, respectively [3–5].

### Robo receptors regulate medial–lateral position of axon pathways in *Drosophila*

In *Drosophila,* the secreted Slit ligand and its Robo receptors regulate midline crossing of axons in the developing embryonic CNS. In addition, two of the three Robo family members in *Drosophila* (Robo2 and Robo3) specify the medial–lateral position of longitudinal axon pathways. The functional specialization of the three *Drosophila* Robos depends on both their differential expression in distinct neuronal subsets and distinct activities of the receptors themselves [6–9]. Previous gene replacement experiments using a knock-in-based approach demonstrated that exons 2-12 of the *Drosophila robo3* gene, along with all of the intervening introns, can be replaced with a non-native cDNA encoding *robo3* without any detectable changes to its expression pattern or function [8]. Equivalent experiments replacing *robo3* with *robo1* or *robo2* cDNAs further demonstrated that each of these paralogs can fully substitute for *robo3* to promote axon pathway formation in intermediate regions of the neuropile in the *Drosophila* embryonic CNS. In contrast, neither *robo1* nor *robo3* can rescue *robo2*-dependent formation of lateral pathways, suggesting that unique structural features of Robo2 specify this role [8]. Although it has been proposed that *Drosophila* Robo receptors regulate lateral position in response to a gradient of Slit originating at the CNS midline, the precise mechanism(s) underlying this activity have not been fully characterized [10].

### Divergent genetic programs in *Drosophila* and *Tribolium*

**Fig. 1:**
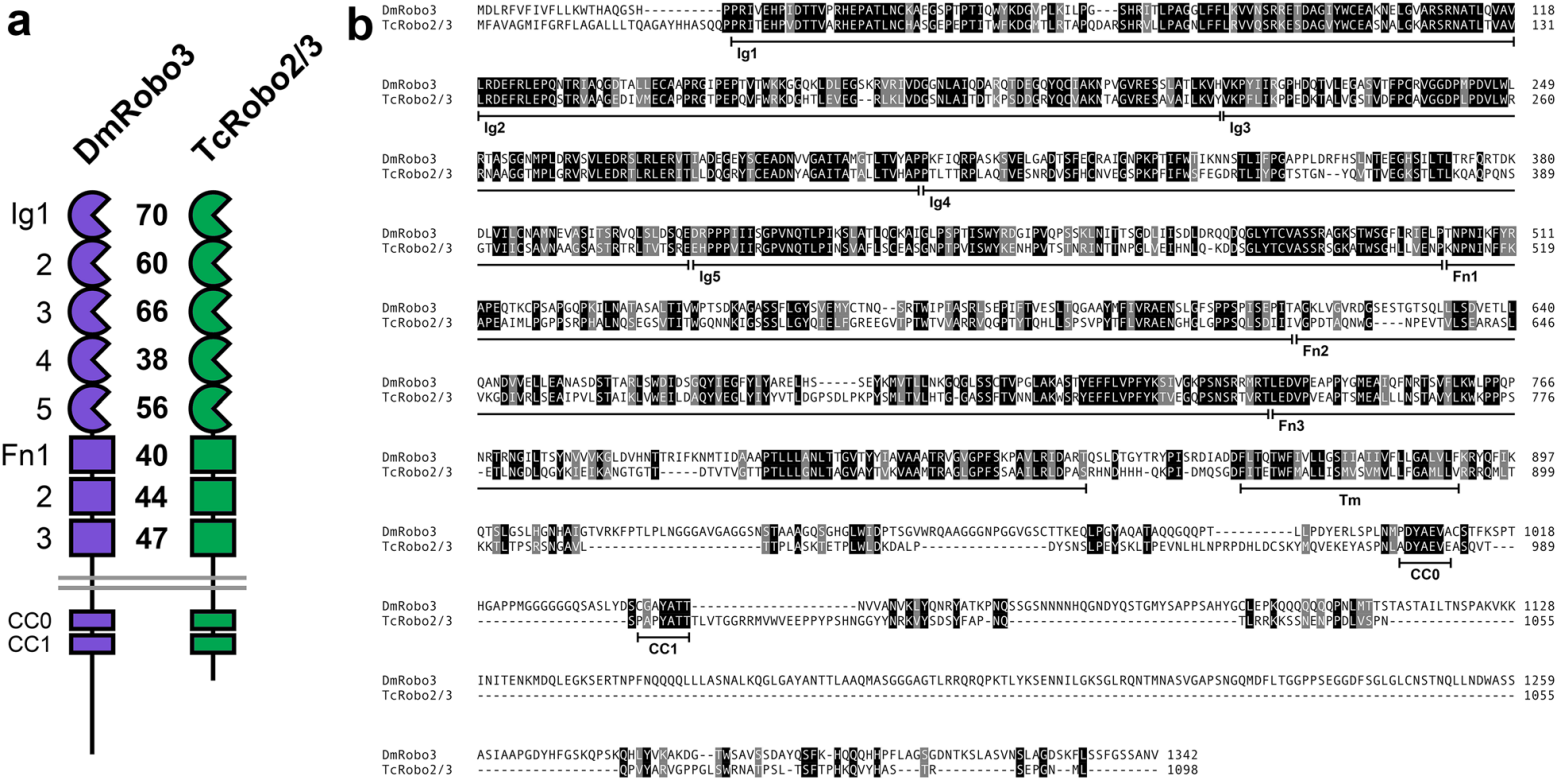
Sequence comparison of *Drosophila* Robo3 and *Tribolium* Robo2/3. **a** Schematic comparison of the two receptors showing conserved domain structure and percent identity between individual ectodomain elements. The highest degree of sequence conservation occurs within the Slit-binding Ig1 domain (70% identity). While both proteins share the evolutionarily conserved CC0 and CC1 motifs, the TcRobo2/3 cytodomain (206 aa) is less than half the length of the Robo3 cytodomain (452 aa). **b** Protein sequence alignment. Structural features are indicated below the sequence. Fn domains have been re-annotated relative to Evans and Bashaw [11] based on revised predictions of beta strand locations. Identical residues are *shaded black*; similar residues are *shaded gray*. *Ig* immunoglobulin-like domain, *Fn* fibronectin type III repeat, *Tm* transmembrane helix, *CC* conserved cytoplasmic motif

While *robo2* and *robo3* have distinct expression patterns and axon guidance roles in *Drosophila*, these two genes are a product of a recent gene duplication and do not exist as separate genes outside of dipteran insects. Instead, most insect groups have retained an ancestral *robo2/3* gene [11, 12]. In the flour beetle *Tribolium castaneum,* this gene (*TcRobo2/3*) appears to combine the activities of *Drosophila robo2* and *robo3* and is required for the formation of both intermediate and lateral axon pathways [11]. The TcRobo2/3 protein exhibits the characteristic ectodomain structure common to most Robo receptors, combining five immunoglobulin-like domains with three fibronectin type III repeats (Fn) within its extracellular portion. TcRobo2/3 shares a high degree of sequence identity with *Drosophila* Robo2 and Robo3 throughout its extracellular portion, with the highest degree of sequence conservation in the Ig domains, especially the Slit-binding Ig1 domain (Fig. 1) [11]. Sequences within the cytoplasmic domain are less well conserved, and this portion of the protein is significantly shorter in TcRobo2/3 than in *Drosophila* Robo3 (206 aa versus 452 aa, respectively). Despite this size difference, the two conserved cytoplasmic (CC) motifs present in *Drosophila* Robo3 are also present in TcRobo2/3. These two motifs, named CC0 and CC1, are conserved in most Robo family members, including *Drosophila* Robo1, Robo2, and Robo3, as well as Robo1 and Robo2 in vertebrates [13].

### CRISPR/Cas9-based trans-species gene replacement

We have previously hypothesized that the ancestor of *robo2* and *robo3* was required for both intermediate and lateral pathway formation, and that this dual activity is retained by the *robo2/3* gene in most insect species (including *Tribolium*), while the activities of *Drosophila robo2* and *robo3* have diverged through subfunctionalization after gene duplication [10, 11]. A prediction of this model is that *TcRobo2/3* should be able to substitute for both *Drosophila robo3* and *robo2* to promote intermediate and lateral pathway formation, respectively. Here, I test this prediction by replacing the *robo3* gene in *Drosophila* with the *robo2/3* gene from *Tribolium* via CRISPR/Cas9-mediated gene replacement. I find that when expressed from the *robo3* locus, TcRobo2/3 protein reproduces the endogenous Robo3 expression pattern in the *Drosophila* embryonic CNS, including proper localization to neuronal axons and distribution on longitudinal axon pathways within intermediate and lateral regions of the neuropile. Further, *TcRobo2/3* is able to substitute for *robo3* to direct two distinct subsets of longitudinal axons to intermediate regions of the neuropile. These results suggest that *Drosophila robo3* and *Tribolium robo2/3* regulate axon pathway formation through an evolutionarily conserved mechanism and demonstrate the utility of CRISPR/Cas9-mediated gene replacement for comparative studies in evolutionary developmental biology.

## Methods

### Molecular biology

#### Construction of robo3^TcRobo2/3^ donor plasmid

The initial *robo3* donor construct was assembled from four PCR fragments via Gibson assembly (New England Biolabs E2611). The four fragments were derived from pBluescript (plasmid backbone; primer pair 417–418), the wild-type *robo3* genomic locus (5′ and 3′ homology regions; primer pairs 501–502 and 505–506), and the *robo3* cDNA (*robo3* coding region; primer pair 503–504). This initial donor construct contained an untagged *robo3* cDNA flanked by BglII sites. To make the HA-tagged *robo3* ^*TcRobo2/3*^ donor, the *robo3* coding sequence was excised with BglII and a 4xHA sequence flanked by BamHI (upstream) and BglII (downstream) sites was cloned into the BglII site, thus retaining a single BglII cloning site immediately downstream of the 4xHA tag. The *TcRobo2/3* coding sequence was amplified by PCR using primers 279 and 280, then digested with BglII, and cloned into the 4× HA-containing donor backbone. The entire donor region including *TcRobo2/3* coding sequence and *robo3* flanking regions was sequenced prior to injection.

#### Construction of robo3 gRNA plasmid

*robo3* gRNA sequences were cloned into the tandem expression vector pCFD4 [14] via PCR using primers 507 and 508, followed by Gibson assembly using the PCR product and BbsI-digested pCFD4 backbone. In both cases, an additional G nucleotide was added to the 5′ end of the gRNA target sequence to facilitate transcription from the U6-1 and U6-3 promoters.

### Genetics

#### Drosophila strains

The following *Drosophila* strains, transgenes, and mutant alleles were used: Canton-S (wild type), *robo3* ^*1*^ [7], *robo3* ^*robo3*^ [8], *robo3* ^*TcRobo2/3*^ (this study), *P{sema2b*-*TauMyc}* [7], *w* ^*1118*^ *; sna* ^*Sco*^ */CyO,P{en1}wg* ^*en11*^ (“*Sco/CyOwg*”). All crosses were carried out at 25 °C.

#### Generation and recovery of CRISPR-modified alleles

The *robo3* gRNA and *robo3* ^*TcRobo2/3*^ homologous donor plasmids were coinjected into *nos*-*Cas9.P* embryos [14] by BestGene Inc. (Chino Hills, CA). Injected individuals (G0) were crossed as adults to *Sco/CyOwg*. Founders (G0 flies producing F1 progeny carrying modified *robo3* alleles) were identified by testing two pools of three F1 females per G0 cross by genomic PCR with primers 229 and 280, which produce a 0.8-kb product only when *TcRobo2/3* sequences are present. From each identified founder, 5–10 F1 males were then crossed individually to *Sco/CyOwg* virgin females. After three days, the F1 males were removed from the crosses and tested by PCR with primers 229 and 280 to determine whether they carried the modified allele. F2 flies from positive F1 crosses were used to generate balanced stocks, and the modified alleles were fully sequenced by amplifying the entire modified locus (approx. 6 kb) from genomic DNA using primers 589 and 590, then sequencing the PCR product after cloning via CloneJET PCR cloning kit (Thermo Scientific). Details of G0 survival, fertility, and modified allele transmission rates are provided in Table 1.

**Table 1:** Survival, fertility, and transmission rates for CRISPR replacement of *robo3*

Injection line	# embryos injected	% (no.) adult G0 survival	% (no.) fertile G0 adults	% (no.) G0 founders	% (no.) HDR F1 progeny
*nos*-*Cas9.P* (BDSC #54591)	300	31 (93/300)	66 (61/93)	25 (5/20)	43 (5/5, 5/10, 2/10, 1/5, 2/5)

*robo3* ^*TcRobo2/3*^ donor and gRNA plasmids were coinjected into *nos*-*Cas9* embryos. Injected individuals (G0) were crossed as adults to *Sco/CyOwg*, and the resulting F1 progeny were screened by PCR for the presence of the expected *robo3* ^*TcRobo2/3*^ allele. Although 61 G0 crosses produced F1 progeny, only 20 of these were screened. The percentage of crosses producing at least one modified allele (founders) is indicated along with the frequency of modified F1 recovered from each founder (average recovery was 43%, range 20–100%). BDSC, Bloomington *Drosophila* Stock Center; HDR, homology-directed repair

### Immunohistochemistry

Drosophila embryo collection, fixation, and antibody staining were carried out as previously described [15]. The following antibodies were used: FITC-conjugated goat anti-HRP (Jackson ImmunoResearch #123-095-021, 1:100), Alexa 647-conjugated goat anti-HRP (Jackson #123-605-021, 1:100), mouse anti-Fasciclin II (Developmental Studies Hybridoma Bank [DSHB] #1D4, 1:100), mouse anti-βgal (DSHB #40-1a, 1:150), mouse anti-Robo3 (DSHB #15H2, 1:100), mouse anti-HA (Covance #MMS-101P-500, 1:1000), rabbit anti-c-Myc (Sigma-Aldrich #C3956, 1:500), Cy3-conjugated goat anti-mouse (Jackson #115-165-003, 1:1000), Alexa 488-conjugated goat anti-rabbit (Jackson #111-545-003, 1:500). Embryos were genotyped using balancer chromosomes carrying lacZ markers. Ventral nerve cords from embryos of the desired genotype and developmental stage were dissected and mounted in 70% glycerol/PBS. Fluorescent confocal stacks were collected using a Leica SP5 confocal microscope and processed by Fiji/ImageJ [16] and Adobe Photoshop software.

## Results

### CRISPR/Cas9-based gene replacement of *Drosophila robo3*

**Fig. 2:**
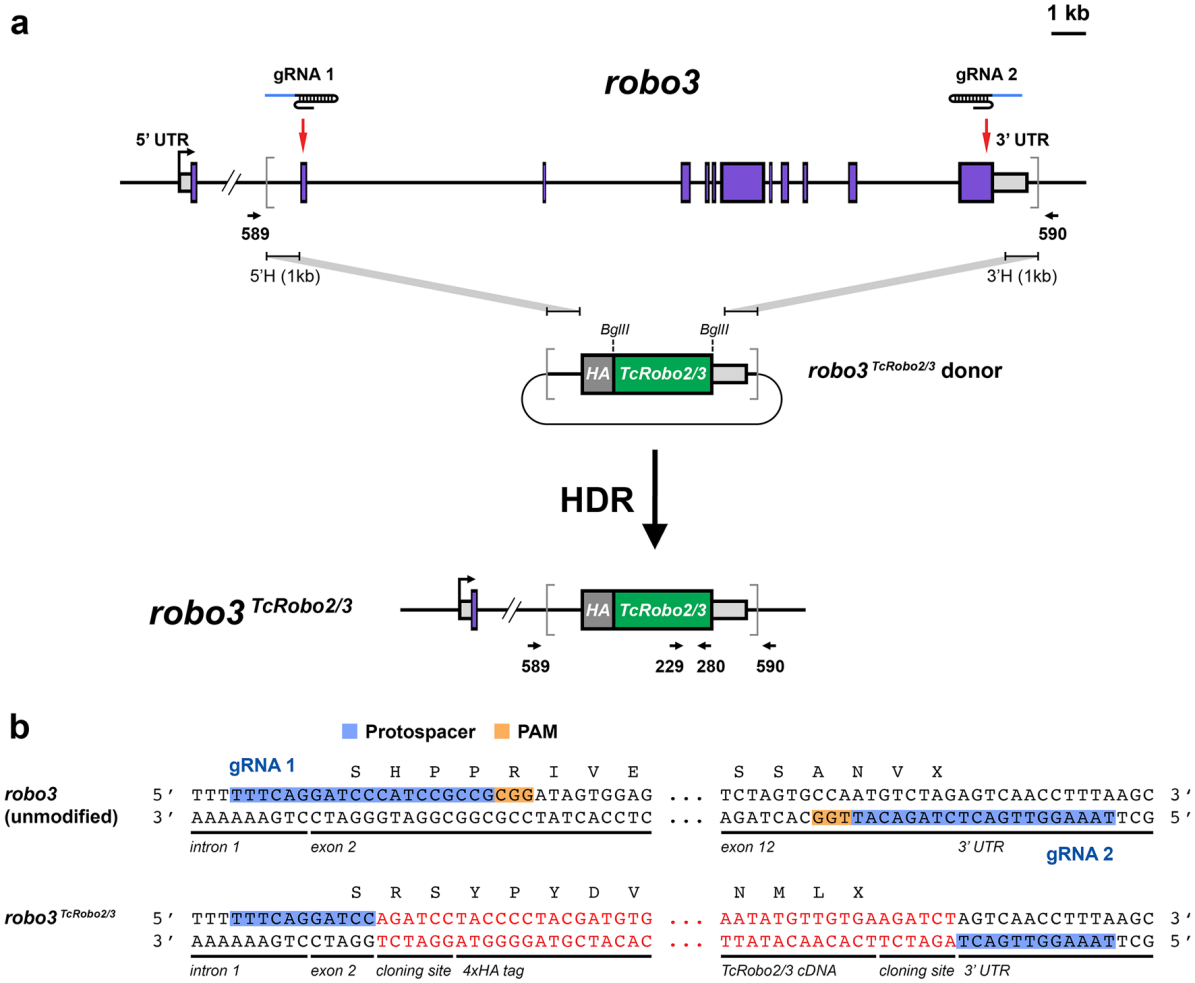
CRISPR-based gene replacement of *robo3.* **a** Schematic of the *robo3* gene showing intron/exon structure and location of gRNA target sites, *robo3* ^*TcRobo2/3*^ homologous donor plasmid, and the final modified *robo3* ^*TcRobo2/3*^ allele. Endogenous *robo3* coding exons are shown as *purple boxes*; 5′ and 3′ untranslated regions are shown as *light gray boxes*. The start of transcription is indicated by the *bent arrow*. Introns and exons are shown to scale, with the exception of the first intron, from which approximately 13 kb has been omitted. *Red arrows* indicate the location of upstream (gRNA 1) and downstream (gRNA 2) gRNA target sites. *Gray brackets* demarcate the region to be replaced by sequences from the donor plasmid. *Arrows* indicate the position and orientation of PCR primers. **b** Partial DNA sequences of the unmodified *robo3* gene and the modified *robo3* ^*TcRobo2/3*^ allele. *Black letters* indicated endogenous DNA sequence; *red letters* indicate exogenous sequence. Both DNA strands are illustrated. The gRNA protospacer and PAM sequences are indicated for both gRNAs. The first five base pairs of *robo3* exon 2 are unaltered in the *robo3* ^*TcRobo2/3*^ allele, and the *robo3* coding sequence beginning with codon H21 is replaced by the HA-tagged *TcRobo2/3* cDNA. The endogenous *robo3* transcription start site, ATG start codon, and signal peptide are retained in exon 1. The PAM sequences and portions of both protospacers are deleted in the modified allele, ensuring that the *robo3* ^*TcRobo2/3*^ donor plasmid and modified *robo3* ^*TcRobo2/3*^ allele are not targeted by Cas9. *UTR* untranslated regions, *5*′*H* 5′ homology region, *3′H* 3′ homology region, *HA* hemagglutinin epitope tag, *gRNA* guide RNA, *HDR* homology-directed repair, *PAM* protospacer adjacent motif

To test whether *Tribolium Robo2/3* can substitute for *Drosophila robo3* to promote axon guidance outcomes in the *Drosophila* embryonic CNS, I used a CRISPR/Cas9-based approach to replace the *robo3* gene with *TcRobo2/3*. Two guide RNAs (gRNAs) targeting exons 2 and 12 were combined with a homologous donor plasmid containing 1-kb flanking regions to induce homology-directed repair, replacing *robo3* exons 2-12 with an HA-tagged *TcRobo2/3* cDNA (Fig. 2). Briefly, a plasmid expressing both *robo3* gRNAs (pCFD4) [14] was injected along with the *robo3* ^*TcRobo2/3*^ donor plasmid into *Drosophila* embryos expressing Cas9 under the control of the germline-specific *nanos* promoter [14], and F1 progeny produced from the injected flies were screened by PCR for the presence of *TcRobo2/3* sequences. Additional PCR screening and DNA sequencing were used to identify correctly modified *robo3* ^*TcRobo2/3*^ loci among the lines recovered from the positive F1 flies. Further details are provided in Methods and Table 1.

### The *robo3*^*TcRobo2/3*^ allele reproduces the Robo3 expression pattern

**Fig. 3:**
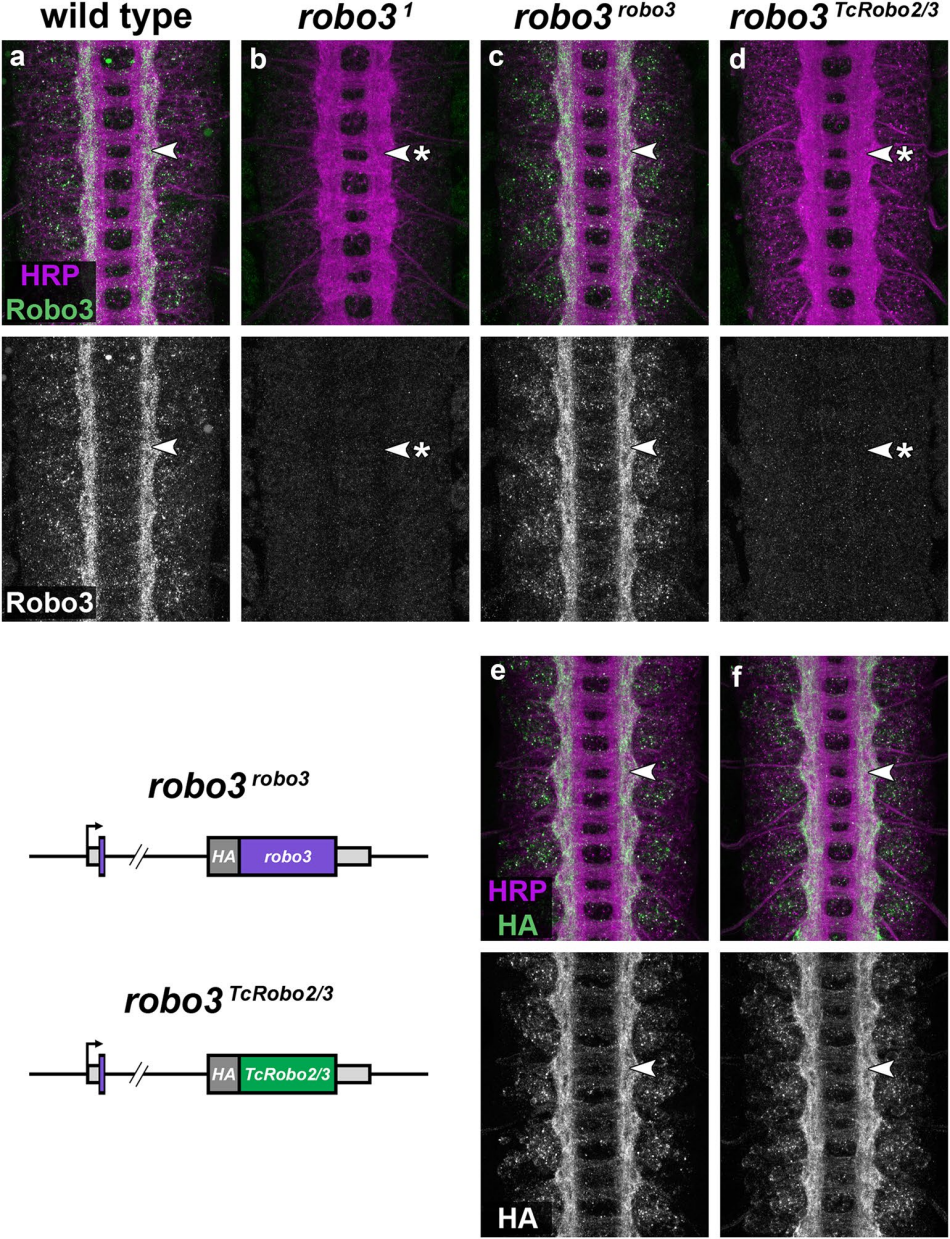
TcRobo2/3 expression reproduces Robo3’s expression pattern in the *robo3* ^*TcRobo2/3*^ allele. **a**–**d** Stage 16 *Drosophila* embryos stained with anti-HRP (*magenta*; labels all axons) and anti-Robo3 (*green*) antibodies. *Lower images* show anti-Robo3 channel alone from the same embryos. In wild-type embryos, endogenous Robo3 protein is detectable on longitudinal axons within the outer two-thirds of the neuropile (**a**, *arrowhead*). Robo3 protein is undetectable in embryos homozygous for the loss of function *robo3* ^*1*^ allele (**b**, *arrowhead* with *asterisk*) [7, 8]. There are no large-scale defects detectable with anti-HRP in the axon scaffold of *robo3* ^*1*^ mutants. In embryos in which the *robo3* gene has been replaced with an HA-tagged *robo3* cDNA, Robo3 protein expressed from the modified locus reproduces its normal expression pattern (**c**, *arrowhead*) [8]. In our CRISPR-modified embryos in which *robo3* has been replaced by *TcRobo2/3*, Robo3 protein is undetectable, consistent with the removal of *robo3* coding sequences (**d**, *arrowhead* with *asterisk*). **e**, **f** Stage 16 embryos stained with anti-HRP (*magenta*) and anti-HA (*green*) antibodies. *Lower images* show anti-HA channel alone from the same embryos. Anti-HA staining in *robo3* ^*robo3*^ embryos detects the Robo3 protein expressed from the modified locus and reproduces the staining pattern seen with anti-Robo3 (**e**, *arrowhead*). In *robo3* ^*TcRobo2/3*^ embryos, the HA-tagged TcRobo2/3 protein reproduces Robo3’s expression pattern and is detectable on longitudinal axons within the lateral two-thirds of the neuropile (**f**, *arrowhead*). Schematics of the two modified *robo3* alleles are shown at *lower left*. The *robo3* ^*robo3*^ allele was generated by Spitzweck et al. [8]

To characterize the *robo3* ^*TcRobo2/3*^ allele, I first compared its expression to that of the endogenous Robo3 protein in various wild-type and modified genetic strains (Fig. 3). In wild-type *Drosophila* embryos, Robo3 expression first becomes detectable on axons in the CNS around stage 14, when pioneering longitudinal axons begin to separate into distinguishable medial and intermediate axon pathways [6, 7, 17, 18]. By stage 16, the mature Robo3 expression pattern has been established: Robo3 protein is expressed on longitudinal axons in the intermediate and lateral regions of the neuropile and is excluded from axons within the medial region closest to the midline (Fig. 3a). In other words, axons expressing Robo3 choose to form or join longitudinal pathways within the lateral two-thirds of the axon scaffold. This is in contrast to Robo1, which is expressed on axons across the entire width of the scaffold (and has no role in lateral positioning), and Robo2, which is restricted to axons within only the most lateral region of the neuropile and promotes longitudinal pathway formation in that region [6, 7].

In embryos homozygous for the strongly hypomorphic/null *robo3* ^*1*^ allele, Robo3 protein is undetectable in the CNS. Although there are longitudinal axon defects caused by the loss of *robo3* (see below), there are no detectable large-scale defects in the axon scaffold in *robo3* ^*1*^ mutants, as assayed by an antibody against horseradish peroxidase (anti-HRP), which labels all of the axons in the CNS (Fig. 3b) [7]. Spitzweck et al. [8] previously reported a modification of the *robo3* gene in which the majority of the *robo3* coding exons and intervening introns were replaced by an HA-tagged *robo3* cDNA (*robo3* ^*robo3*^). In *robo3* ^*robo3*^ homozygous embryos, the Robo3 protein expressed from the modified locus precisely reproduces Robo3’s normal expression pattern (Fig. 3c), and the same pattern can be seen when staining against the N-terminal HA epitope tag present in the modified allele (Fig. 3e) [8].

In the *robo3* ^*TcRobo2/3*^ allele, the anti-Robo3 antibody fails to detect any protein in the embryonic CNS, consistent with the replacement of the *robo3* coding region with that of *TcRobo2/3* (Fig. 3d). Anti-HA staining reveals that expression of the TcRobo2/3 protein in homozygous *robo3* ^*TcRobo2/3*^ embryos is indistinguishable from that of Robo3 in wild-type or *robo3* ^*robo3*^ embryos (Fig. 3f). In *robo3* ^*TcRobo2/3*^ embryos, TcRobo2/3 protein is localized to axons within the intermediate and lateral regions of the neuropile and is excluded from both commissural (midline crossing) axon segments and longitudinal axons in the medial neuropile. No appreciable levels of TcRobo2/3 appear to accumulate within the cell bodies of normally *robo3*-expressing neurons, or on the cell body plasma membrane, consistent with proper subcellular localization of the *Tribolium* Robo2/3 protein in this heterologous context. These observations indicate that the TcRobo2/3 protein is properly translated and trafficked within *Drosophila* neurons, and suggest that axons that would normally select intermediate or lateral pathways under conditions of wild-type Robo3 expression make equivalent pathway choices when TcRobo2/3 is expressed in its place.

### *TcRobo2/3* can promote intermediate axon pathway formation in the absence of *robo3*

**Fig. 4:**
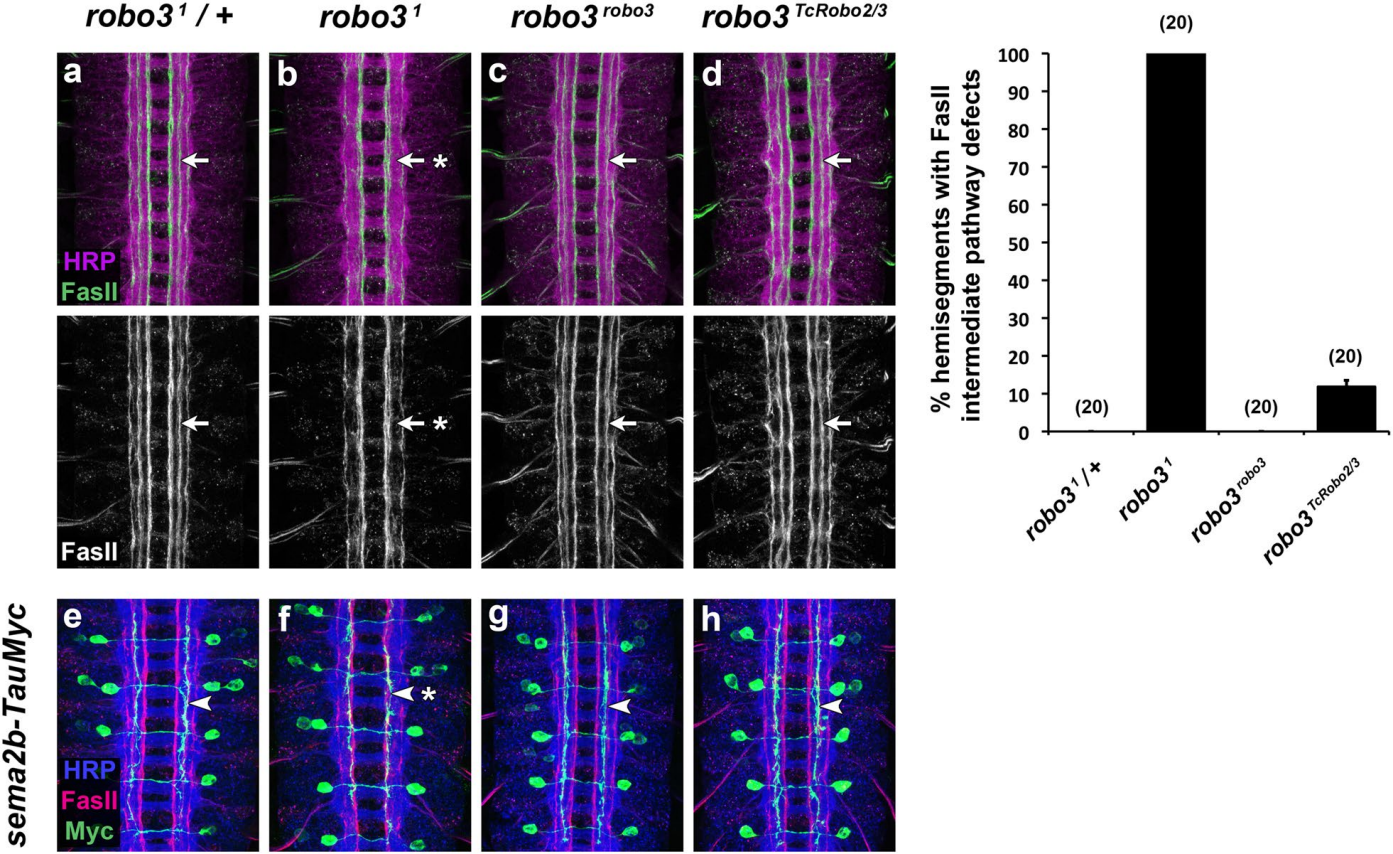
*Tribolium* Robo2/3 can substitute for *Drosophila* Robo3 to promote axon pathway formation in *Drosophila* embryos. **a**–**d** Stage 16 *Drosophila* embryos stained with anti-HRP (*magenta*) and anti-FasII (*green*) antibodies. *Lower images* show anti-FasII channel alone from the same embryos. In wild-type embryos, FasII-positive axons form three distinct longitudinal pathways on either side of the midline, one each in the medial, intermediate, and lateral zones of the neuropile. The intermediate FasII pathway is distinct from the medial and lateral pathways in every hemisegment in wild-type embryos (**a**, *arrow*). In *robo3* ^*1*^ embryos, FasII-positive axons that normally form the intermediate pathway are displaced medially, and the intermediate pathway fails to form (**b**, *arrow* with *asterisk*). Intermediate pathways form correctly in embryos in which the *robo3* gene is replaced with a *robo3* cDNA (**c**, *arrow*). When *robo3* is replaced with a *TcRobo2/3* cDNA, intermediate pathways form correctly in over 88% of hemisegments (**d**, *arrow*), indicating that *TcRobo2/3* can substitute for *robo3* to promote axon pathway formation in the intermediate region of the neuropile. *Bar graph* shows quantification of intermediate FasII pathway defects in the genotypes shown in **a**–**d**. *Error bars* indicate standard error of the mean. Number of embryos scored for each genotype is indicated in *parentheses*. **e**–**h** Embryos carrying the *sema2b*-*TauMyc* transgene and stained with anti-HRP (*blue*), anti-FasII (*red*), and anti-Myc (*green*) antibodies. The *sema2b*-*TauMyc* transgene labels the cell bodies and axons of 2–3 neurons per hemisegment in abdominal segments A4–A8. These axons normally project across the midline and then extend anteriorly in the intermediate region of the neuropile (**e**, *arrowhead*). In *robo3* ^*1*^ embryos, these axons are displaced medially (**f**, *arrowhead* with *asterisk*), but their normal intermediate position is restored in both *robo3* ^*robo3*^ (**g**, *arrowhead*) and *robo3* ^*TcRobo2/3*^ embryos (**h**, *arrowhead*)

 To more closely examine the formation of longitudinal axon pathways in *robo3* ^*TcRobo2/3*^ embryos, I used an antibody against the cell adhesion molecule Fasciclin II (FasII) to label a subset of longitudinal axon pathways in these embryos (Fig. 4) [19]. In the ventral nerve cord of wild-type *Drosophila* and *Tribolium* embryos, at least eight distinct FasII-positive longitudinal axon pathways form at stereotypical dorsal–ventral and medial–lateral positions within the neuropile of each abdominal hemisegment [1, 11]. In *Drosophila, robo3* is required for formation of FasII-positive axon pathways in the intermediate region of the neuropile, while *robo2* is required for formation of lateral pathways [6, 7]. In *Tribolium, TcRobo2/3* is required for proper positioning of both intermediate and lateral pathways and thus appears to combine the activities that in *Drosophila* are divided between *robo2* and *robo3* [11]. We previously hypothesized that this represents subfunctionalization between *robo2* and *robo3* in dipterans, and predicted that *TcRobo2/3* might therefore retain the ability to rescue both *robo2*’s and *robo3’*s lateral positioning roles in *Drosophila* [10, 11].

To determine whether TcRobo2/3 can act equivalently to Robo3 to promote axon pathway formation in intermediate regions of the *Drosophila* embryonic CNS, I used anti-FasII to examine the development of intermediate FasII-positive axon pathways in the various genetic backgrounds described above (Fig. 4). In *robo3* ^*1*^ mutant embryos, FasII-positive intermediate axon pathways fail to form in their proper position, instead shifting closer to the midline and merging with pathways in the medial zone (Fig. 4b) [6, 7]. This defect appears in 100% of hemisegments in *robo3* ^*1*^ mutants and is fully rescued when the *robo3* locus is replaced with an HA-tagged wild-type *robo3* cDNA (Fig. 4c) [8]. I found that proper formation of intermediate axon pathways is also restored in over 88% of hemisegments when *robo3* is replaced by *TcRobo2/3* (Fig. 4d), indicating that *TcRobo2/3* can substitute for *Drosophila robo3* to promote axon pathway formation in the fly embryonic CNS.

While FasII-positive longitudinal axon pathways are the most commonly used marker for *robo3*-dependent medial–lateral positioning in the *Drosophila* CNS, other axons rely on *robo3* for their proper positioning within the intermediate neuropile, including chordotonal sensory axons and FasII-negative longitudinal axons [6, 7, 20]. To ask whether *TcRobo2/3*’s ability to substitute for *robo3* also applies to other neuronal subsets in the *Drosophila* embryonic CNS, I examined a subset of neurons labeled by the *sema2b*-*TauMyc* marker, which relies on a cloned promoter fragment from the semaphorin gene *sema2b* to express the microtubule-binding TauMyc fusion protein in a subset of *sema2b*-expressing longitudinal axons [7]. These *sema2b*-positive axons do not express FasII, and they cross the midline to form a contralateral longitudinal pathway in the intermediate neuropile at an earlier timepoint than the FasII-positive axons examined above [5] (Fig. 4e). Consistent with previous reports, I found that the *sema2b* axons are shifted to a medial position in *robo3* ^*1*^ mutants, but select their normal intermediate position in *robo3* ^*robo3*^ embryos [7, 8] (Fig. 4f, g). In *robo3* ^*TcRobo2/3*^ embryos, the *sema2b* axons also select their normal intermediate position, indicating that *TcRobo2/3* can substitute for *robo3* to direct these axons to the intermediate region of the neuropile. Together, these results demonstrate that *Tribolium Robo2/3* can act equivalently to *Drosophila robo3* to specify an intermediate lateral position in at least two distinct sets of interneurons in the fly embryonic CNS.

## Discussion

In this paper, I have used a CRISPR/Cas9-based gene replacement approach to directly examine the evolutionary conservation of axon guidance activities within the Robo family of axon guidance receptors. I have replaced the *Drosophila melanogaster robo3* gene with its ortholog from the flour beetle *Tribolium castaneum, TcRobo2/3.* I compared the expression of TcRobo2/3 protein in this modified background with the normal expression of Robo3 in wild-type embryos and examined Robo3-dependent guidance of two distinct subsets of longitudinal axons in the ventral nerve cord of wild-type, *robo3* mutant, and *robo3* ^*TcRobo2/3*^ gene replacement embryos. My results suggest that the mechanism(s) by which *robo3* and *TcRobo2/3* regulate axon guidance outcomes in the *Drosophila* and *Tribolium* embryonic CNS, respectively, are evolutionarily conserved. In addition, the gene replacement approach described here should be broadly applicable to evo-devo studies of other genes in *Drosophila* and other insects.

### Evolutionary conservation of Robo3-dependent axon guidance mechanisms

Three lines of evidence suggest that TcRobo2/3 can act equivalently to Robo3 to guide *Drosophila* axons: (1) The subset of longitudinal axons that normally express Robo3 are still localized to the lateral two-thirds of the neuropile when they express TcRobo2/3 instead of Robo3; (2) FasII-positive axon pathways in the intermediate neuropile form in their proper location when *robo3* is replaced by *TcRobo2/3;* and (3) *sema2b*-positive longitudinal axons also select their correct intermediate position when *robo3* is replaced by *TcRobo2/3.* These observations, together with a previous report that RNAi-mediated knockdown of *TcRobo2/3* disrupts intermediate pathway formation in the *Tribolium* embryonic CNS [11], suggest that the mechanism by which *Drosophila* Robo3 promotes longitudinal pathway formation is evolutionarily conserved in *Tribolium*, where it is performed by TcRobo2/3. CRISPR approaches have recently been used successfully in *Tribolium* [21], so it should be possible to perform the complementary experiments by replacing *TcRobo2/3* with *Drosophila robo3.* In this case, *robo3* should be able to replace the function of *TcRobo2/3* to promote intermediate pathway formation, but the lateral pathways (which presumably depend on a *robo2*-like activity of *TcRobo2/3*) may not form properly.

The observation that TcRobo2/3 can fully substitute for Robo3 to regulate intermediate pathway formation in the *Drosophila* embryonic CNS suggests that any sequences that are necessary for this role in Robo3 are conserved in TcRobo2/3. Notably, the highest degree of sequence conservation between these two proteins occurs within the extracellular Ig1 and Ig3 domains (Fig. 1; 77 and 66% identity, respectively); these two domains have also been implicated in *Drosophila* Robo2 in its role in promoting lateral axon pathway formation [9]. While it is not yet known whether Robo2 and Robo3 regulate longitudinal pathway formation via the same mechanism, there are clearly sequences within Robo2 that are not conserved in Robo3 that are essential for guidance of lateral axons [8]. By comparing the conservation and divergence of sequences within *Drosophila* Robo2, *Drosophila* Robo3, and *Tribolium* Robo2/3, it may be possible to identify individual amino acids or short sequence motifs that distinguish between the functions of these proteins.

Both cytoplasmic CC sequences (CC0 and CC1) are conserved in Robo3 and Robo2/3, but there is little conservation in the cytodomain outside of these motifs (Fig. 1). Notably, both CC0 and CC1 are also present in *Drosophila* Robo1 and Robo2, which can both substitute for Robo3 to promote intermediate pathway formation [8]. Thus, if Robo3 acts as a canonical Slit receptor to regulate longitudinal axon guidance, it is likely that this role is dependent on its CC0 and/or CC1 motifs.

### CRISPR/Cas9-mediated gene replacement for studies in evolutionary developmental biology

The gene replacement approach described here has several advantages for both structure–function studies and evo-devo functional comparisons of orthologs from different species. First, replacing multiple coding exons in the endogenous gene with a single cDNA sequence allows for epitope tagging of the modified locus in addition to replacing the entire coding region with a foreign sequence or a modified cDNA carrying engineered deletions, additions, or sequence swaps. For multi-exon genes, this eliminates the need to replace multiple exons individually. Second, the endogenous promoter and translational start site remain unmodified, minimizing effects on gene expression. Third, using two gRNA targets that flank the majority of the gene should allow recovery of a null deletion allele via non-homologous end joining (NHEJ) in addition to the HDR-mediated gene replacement allele. This would be of particular benefit for genes for which a null allele is not currently available. Finally, this approach allows the same gRNA plasmid and donor backbone to be used for multiple gene replacement experiments, minimizing troubleshooting and optimization for each subsequent experiment. We have used the same donor construct backbone to replace *robo3* with modified versions of itself and other *Drosophila robo* genes, and have also recovered a precise deletion of all sequences between the *robo3* gRNA targets (A. Carranza and T.A.E., unpublished). A similar approach should be applicable to other genes assuming that, like all three *Drosophila robo* genes, the introns that would be removed by the cDNA replacement are dispensable for proper expression of the gene to be modified.

## Conclusions

Here I have described a strategy for CRISPR/Cas9-based trans-species gene replacement in *Drosophila* and used this strategy to demonstrate the evolutionary conservation of axon guidance mechanism(s) between the *Drosophila robo3* and *Tribolium robo2/3* genes. When expressed from the *robo3* locus, TcRobo2/3 protein is properly translated and localized to neuronal axons in the *Drosophila* embryonic CNS and can guide developing axons to pathways in the intermediate region of the neuropile in an equivalent manner to *Drosophila* Robo3. The approach described here should be generally applicable to other genes and developmental contexts and will be useful to interrogate the evolutionary conservation or divergence of additional axon guidance mechanisms in insects.

## Abbreviations

CNS: central nervous system
Robo: Roundabout
Sema: semaphorin
FasII: Fasciclin II
CRISPR: clustered regularly interspaced short palindromic repeats
HDR: homology-directed repair
gRNA: guide RNA
PAM: protospacer adjacent motif
Ig: immunoglobulin-like domain
Fn: fibronectin type III repeat
Tm: transmembrane helix
CC: conserved cytoplasmic motif
UTR: untranslated region
5′H: 5′ homology region
3′H: 3′ homology region
HA: hemagglutinin epitope tag
HRP: horseradish peroxidase
BDSC: Bloomington *Drosophila* Stock Center
